# Supplementary material for: Public conservation connection and support between ocean and terrestrial systems in the United States
Source: PLoS One. 2024 Jul 25;19(7):e0307431. doi: 10.1371/journal.pone.0307431 (PMC11271940; doi:10.1371/journal.pone.0307431)
Supplement: S1 File — (PDF) [file pone.0307431.s001.pdf]

Supporting Information

Public conservation connection and support between terrestrial and ocean systems in the United States

Halley E. Froehlich, Darien D. Mizuta, Jono R. Wilson

S1 Table. Survey questions. Used in the descriptive and Random Forest (RF) analysis. If response options differed between the terrestrial and marine surveys they are provided in the respective categories. Questions used as responses in the RF models are highlighted in *yellow*.

Analysis	#	Questions	Terrestrial	Ocean	Scoring
Descriptive	Q6	**Which of the following environmental issues are you aware of?**	• *Deforestation and land use change is causing terrestrial (on-land) biodiversity to decrease*<br>• *Rising average temperatures causing more extreme weather events*<br>• *Increasing levels of household waste*<br>• *Toxic industrial waste causing human health risks*<br>• *Agricultural soil degradation*<br>• *None*	• *Overfishing / Ocean biodiversity is decreasing*<br>• *Melting polar ice caps causing rising sea levels*<br>• *Plastic waste ending up in the ocean*<br>• *Oil spills and industrial waste being dumped into the ocean*<br>• *Ocean acidification*<br>• *None*	1=YES 0=NO
Descriptive/RF	Q8	**In your opinion, is there a need for active conservation efforts in each of the following environments? If so, what level of active conservation effort does each need? (sum and normalize)**	• *Coastal zone, beaches & nearshore ocean habitats*<br>• *Open ocean*<br>• *Freshwater lakes & rivers*<br>• *Forests/woodlands*<br>• *Open plains/grasslands/prairies*<br>• *Mountains*		0= None: This environment doesn't need any active conservation efforts;1 = Minimal; 2 = Moderate ; 3 - Significant; 4 = Major: This environment will collapse without major active conservation effort
Descriptive	Q9	**Imagine that you are in control of a $1 billion dollar budget for environmental conservation programs. The budget needs to be split between ocean & marine vs terrestrial (on-land) environments and wildlife. In your opinion, what percent of the budget would you put towards ocean & marine conservation OR terrestrial (on-land) conservation?**			0-100%
Descriptive/RF	Q10	**Please rate what you feel best matches the current health of terrestrial (on-land) wildlife OR ocean fish and wildlife in and around the United States.**			1=Very bad; 2=Bad; 3=I don't know / Neutral; 4=Good; 5=Very good
Descriptive/RF	Q11	**Please rate what you feel best matches the current health of terrestrial (on-land) OR ocean habitats in and around the United States**			1=Very bad; 2=Bad; 3=I don't know / Neutral; 4=Good; 5=Very good
Descriptive	Q19	**Please read the following description of a conservation project. Based on the description, do you feel that the conservation project was essential and effective?**	*Worldwide forests have suffer from deforestation and have been severely degraded. Since 2008, a reforestation initiative is reforesting by planting trees in the US (and internationally). Tress clean the air, provide habitat to other species, and contribute to clean water and mild climates.*<br><br>*In the US, longleaf and shortleaf pines, hardwood, conifers and others are being planted with the help of stakeholders and local community spanning areas of regions such as the South Central, South Atlantic, and Great Lakes.*	*Over the last 100 years, oyster reefs in New York Harbor declined and eventually collapsed. Oysters improve water quality, reefs provide habitat for other species and are storm barriers.*<br><br>*In 2014, restoration began recycling empty oyster shells. In total 47 million live oysters were restored with 1.6 million pounds of collected shells, with help from stakeholders and the local community.*	1=Strongly disagree that the project was essential and effective; 2=Somewhat disagree; 3=Neutral; 4=Somewhat agree; 5=Strongly agree that the project was essential and effective
Descriptive	Q20	**Conservation practitioners use several scientifically informed approaches for helping at-risk natural habitats and wildlife species. In your opinion, how important do you think each of the following conservation projects is?**	• *Creation of protected areas/ parks*<br>• *Captive breeding of threatened species for reintroduction into nature*<br>• *Relocating species from one degraded habitat to another suitable one*<br>• *Enforcement of rules and regulations*<br>• *Environmental education*<br>• *Captive breeding for supplementing natural stocks for hunting*	• *Creation of protected areas/ parks*<br>• *Captive breeding of threatened species for reintroduction into nature*<br>• *Relocating species from one degraded habitat to another suitable one*<br>• *Enforcement of rules and regulations*<br>• *Environmental education*<br>• *Captive breeding for supplementing natural stocks for fishing*	1=Useless and not important; 2=Not very useful or important; 3=Neutral; 4=Somewhat useful and important; 5=Very useful and important
Descriptive/RF	Q21	**In your opinion, who should be responsible for funding terrestrial (on-land) OR ocean conservation projects in the United States? Please select all the options that you agree with.**	• *Public and/or private investment incentivized by government regulation*<br>• *Government agencies and/or public-private partnerships*<br>• *Individual voluntary donations to conservation nonprofit organizations*<br>• *Private market mechanisms (e.g. payment for ecosystem services)*<br>• *Companies and corporations with environmental responsibility programs*<br>• *Companies whose products damage ocean environments*<br>• *I don't think anyone should be responsible for funding*		1=YES 0=NO
RF	Q4	**Please select which, if any, of the following activities that you enjoy doing for recreation or as a hobby?**	*Fishing, Snorkeling, Boating/sailing, hunting, hiking, camping, none of the above*		1=YES 0=NO
RF	Q4	**Total number of activities**	*Summed Q4*		0-6
RF	Q7	**Take a moment to think about these environmental issues. Who do you feel is impacted by them? (sum and normalize)**	• *Deforestation and land use change is causing terrestrial (on-land) bio-diversity to decrease*<br>• *Rising average temperatures causing more extreme weather events*<br>• *Increasing levels of household waste*<br>• *Toxic industrial waste causing human health risks*<br>• *Agricultural soil degradation*<br>• *None of the above*	• *Overfishing / Ocean biodiversity is decreasing*<br>• *Melting polar ice caps causing rising sea levels*<br>• *Plastic waste ending up in the ocean*<br>• *Oil spills and industrial waste being dumped into the ocean*<br>• *Ocean acidification*<br>• *None of the above*	1= Nobody is impacted by this environmental issue; 2 = Other people are impacted by this, but nobody that I know; 3 = People in my community are impacted by this, but not me; 4= I am impacted by this
RF	Q13	**Please rate how concerned you are personally about the current health of ocean fish & wildlife and ocean habitats OR terrestrial (on-land) wildlife and habitats in the United States?**			*1= Very unconcerned; 2 = Unconcerned 3 = Neutral; 4 = Concerned; 5 = Very concerned*
RF	Q14	**Which, if any, of the following activities have you done in the last two years?**	• *Voted for a politician based on their environmental proposals*<br>• *Donated money to an environmental non-profit or charity*		1=YES 0=NO
RF	Q15	**In your opinion, how effective are each of these activities towards improving the health of ocean habitats and ocean fish OR wildlife/ terrestrial (on-land) habitats and wildlife?**	*Voting for a politician based on their environmental proposals*		1=YES 0=NO
RF	Q16	**Have you personally participated in any environmental conservation efforts?**			1=YES 0=NO
RF	Q18	**Are you aware of any conservation organizations or projects that work to protect at-risk ocean habitats or ocean fish and wildlife OR terrestrial (on-land) habitats or wildlife?**			Open; coded 1=YES 0=NO

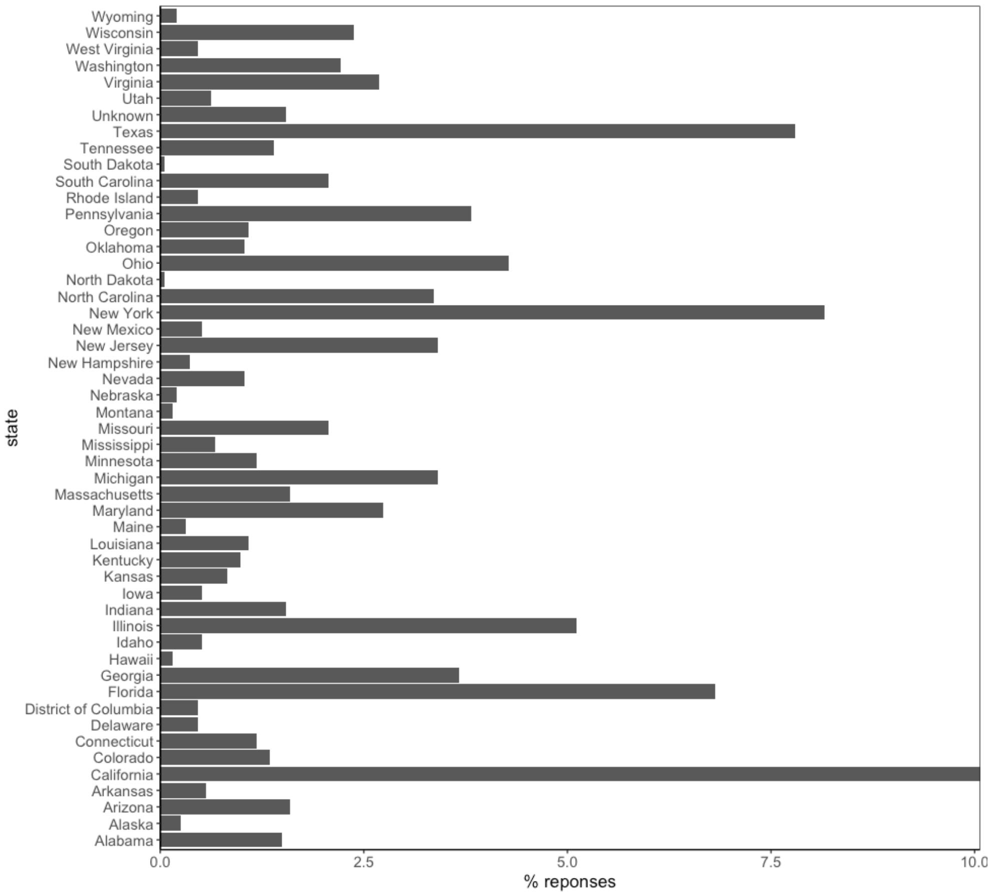

S1 Fig. Geographic distribution. Percent of responses per state. Only Vermont was not represented in the study.

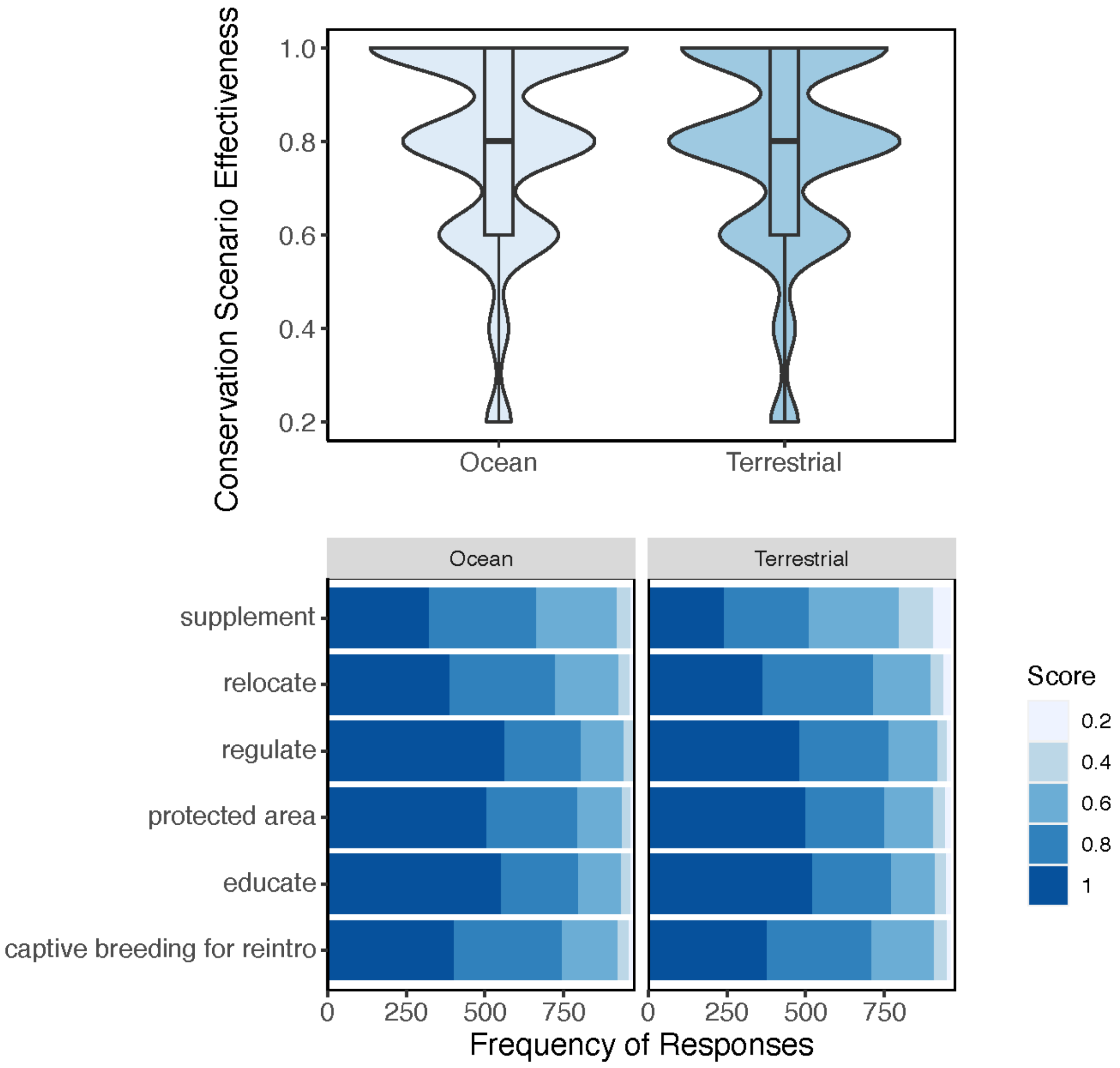

S2 Fig. Conservation interventions. Range and average response of effectiveness of a real-word scenario of oyster restoration versus reforestation (*top panel*) and the frequency of responses scoring the importance of potential conservation approaches (*bottom panel*).

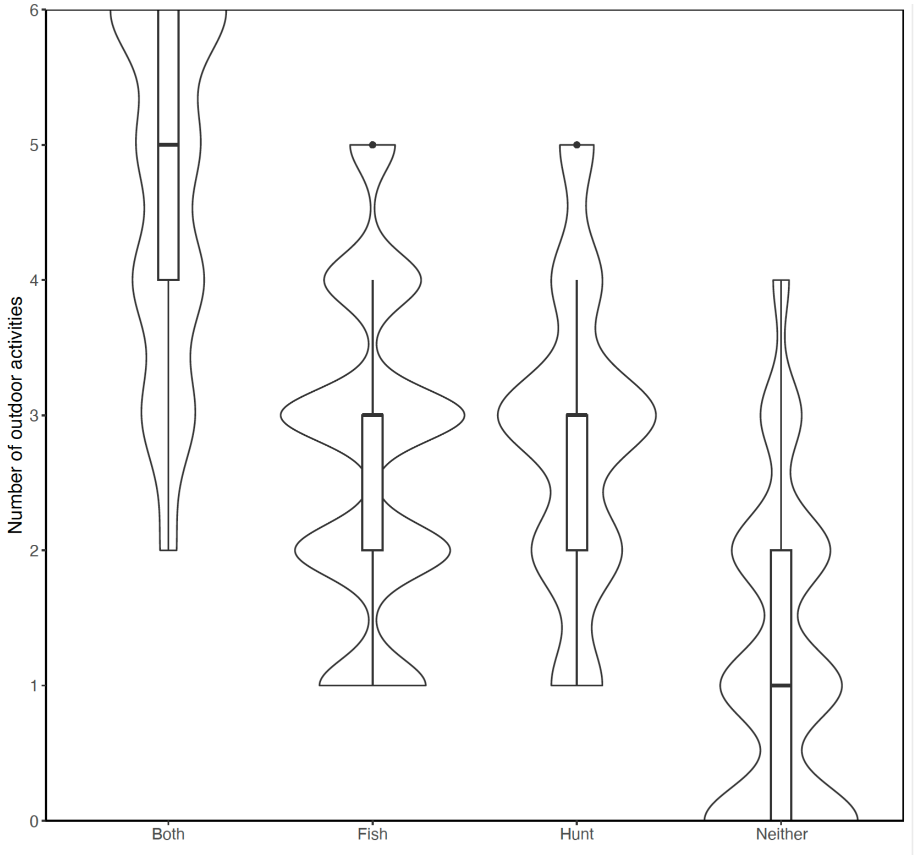

S3 Fig. Association of activities. Average and range of responses of the total number of activities of people who fish, hunt, do both, or do neither.
