# Supplementary material for: Public conservation connection and support between ocean and terrestrial systems in the United States
Source: PLoS One. 2024 Jul 25;19(7):e0307431. doi: 10.1371/journal.pone.0307431 (PMC11271940; doi:10.1371/journal.pone.0307431)
Supplement: Attachment — Submitted filename: PONE-D-24-10931_comments.docx [file pone.0307431.s002.docx]

PONE-D-24-10931

It is suggested to use more results and discussion in the abstract of the article. In the current format, the focus of the abstract is on the introduction

INTRODUCTION

Line 60: You wrote "most US federal policies for conservation are rooted in land-based protections, while ocean-based efforts – mostly passive approaches of marine protected areas...."It is better to mention after this part what reasons they had for paying more attention to the protection of the land than the seas and what factors cause governments and organizations to pay more attention to the protection of the land than the seas.

It is suggested to emphasize the point that different parts of an ecosystem are related to each other and if we consider the earth as a meta-ecosystem, the oceans and land are considered to be part of a single ecosystem, so the emphasis on the mutual effects of land and seas on Each other shows the need to protect both.

Line 70: Your study is based on the dominance hypothesis, so you should first give a brief description of this hypothesis so that it will be easier for the readers of the article to understand the rest of the content.

Line 76: Considering your reference please add some examples for sentence "Education or familiarity with conservation (i.e., conservation literacy) or sustainability activities may play a role in the value of conservation action"

METHODS:

Were the questions used in the questionnaire selected from a specific source?

There are many questions for such studies. What was your criteria for choosing these questions?

Would you please send the codes used for statistical tests in R.

Why did you choose the random forest model for data analysis?

The random forest model suffer from overfitting problem and the output is correlated highly to the input data. How did you over this problem in your analysis?

In the studies that we measure the public opinion and the needs of the society, we must pay attention to the point in which the time and economic conditions of this study are conducted. Have you considered the effect of the time of the survey on the type of response of the society to the questionnaire?

DISCUSSION:

Line 351: Keep in mind that what you have reviewed and studied are the results of questionnaires answered by different groups of people in the community, but what is considered in government and scientific societies for budget allocation and management actions is based on scientific research and projects and precise environmental measurements.

"the oceans and marine animals garnering slightly more concern and support than terrestrial systems and organisms but most global assessments report a greater biodiversity crisis on land" Please explain the reasons of this difference in the views of the public and scientific societies

In general, what is mentioned in the discussion is very similar to the results section of the article, and in fact, in most parts, the results are mentioned without addressing the reasons and interpretation. I recommend rewriting the discussion.

The sample size (N = 1,973) of this study is small, therefore you cannot generalize the results of this study to the entire society, so be careful in choosing words and interpreting the results.

The economic dependence of people on the sea or land ecosystem has a great impact on answering the questions, and this is unfortunately not considered in the interpretation of the results.

Finally, in discussion section, I recommend to use the results of this paper beside broader studies to better comprehend of society view in the case of prioritizing in biodiversity conservation in terrestrial versus ocean
